# Trees as net sinks for methane (CH_4_) and nitrous oxide (N_2_O) in the lowland tropical rain forest on volcanic Réunion Island

**DOI:** 10.1111/nph.17002

**Published:** 2020-11-18

**Authors:** Katerina Machacova, Libor Borak, Thomas Agyei, Thomas Schindler, Kaido Soosaar, Ülo Mander, Claudine Ah‐Peng

**Affiliations:** ^1^ Global Change Research Institute of the Czech Academy of Sciences Belidla 986/4a Brno CZ‐60300 Czech Republic; ^2^ Department of Geography Institute of Ecology & Earth Sciences University of Tartu 46 Vanemuise Tartu EST‐51014 Estonia; ^3^ UMR PVBMT Université de La Réunion 7 chemin de l’IRAT Saint‐Pierre, La Réunion F‐97410 France

**Keywords:** basaltic lava flows, cryptogams, methane flux, nitrous oxide flux, soil, tree stem, tropical lowland rain forest, uptake

## Abstract

- Trees are known to emit methane (CH_4_) and nitrous oxide (N_2_O), with tropical wetland trees being considerable CH_4_ sources. Little is known about CH_4_ and especially N_2_O exchange of trees growing in tropical rain forests under nonflooded conditions.
- We determined CH_4_ and N_2_O exchange of stems of six dominant tree species, cryptogamic stem covers, soils and volcanic surfaces at the start of the rainy season in a 400‐yr‐old tropical lowland rain forest situated on a basaltic lava flow (Réunion Island). We aimed to understand the unknown role in greenhouse gas fluxes of these atypical tropical rain forests on basaltic lava flows.
- The stems studied were net sinks for atmospheric CH_4_ and N_2_O, as were cryptogams, which seemed to be co‐responsible for the stem uptake. In contrast with more commonly studied rain forests, the soil and previously unexplored volcanic surfaces consumed CH_4_. Their N_2_O fluxes were negligible.
- Greenhouse gas uptake potential by trees and cryptogams constitutes a novel and unique finding, thus showing that plants can serve not only as emitters, but also as consumers of CH_4_ and N_2_O. The volcanic tropical lowland rain forest appears to be an important CH_4_ sink, as well as a possible N_2_O sink.

## Introduction

Covering an area of *c.* 1730 million ha, tropical forests comprise 45% of the global forested area (D’Annunzio *et al*., 2017). The most extensive tropical forest type is tropical rain forest, which encompasses almost 60% of all tropical forest area (Shvidenko *et al*., 2005). These forests are found predominantly in South America, Africa and Asia, and are characterized as closed‐canopy evergreen broadleaf forests with minimum annual temperature and precipitation of 25°C and 1500 mm, respectively (Richards, 1996). In general, tropical forests (including all kinds of forest types) constitute important carbon (C) stock (428 Gt‐C in vegetation and soil) and C sink (–0.37 t‐C ha^−1^ yr^−1^; Dalal & Allen, 2008). Furthermore, they are considered to be a natural source of nitrous oxide (N_2_O), and a natural sink and source of methane (CH_4_), both of which are important greenhouse gases (GHG) with global warming potential of 265–298 and 28–36 over 100 yr, respectively (Myhre *et al*., 2013). Net N_2_O emission from tropical forest soils is estimated to be 4.76 kg ha^−1^ yr^−1^, whereas net soil CH_4_ consumption, without considering possible canopy fluxes from trees (Keppler *et al*., 2006), seems to be − 3.86 kg ha^−1^ yr^−1^ (Dalal & Allen, 2008). In the case of CH_4_, soils of tropical rain forests growing under submerged conditions (e.g. Amazonia) can be substantial CH_4_ sources as well, as a consequence of the prevailing anaerobic conditions required for CH_4_ production (Pangala *et al*., 2017). Tropical forest soils have the highest N_2_O emission potential among natural forest ecosystems (Dalal & Allen, 2008), and therefore tropical forests play an essential role in global N_2_O inventories.

Nitrous oxide is naturally produced in soils through a wide range of nitrogen (N) turnover processes having different soil water content optima, including aerobic nitrification, anaerobic denitrification, and also dissimilatory nitrate reduction to ammonium in suboxic conditions (Espenberg *et al*., 2018). The denitrification processes are the only processes known to reduce N_2_O to dinitrogen (N_2_) (Smith *et al*., 2003). By contrast, CH_4_ is produced by strictly anaerobic methanogenesis in water‐saturated soils and is oxidized by methanotrophic bacteria (Smith *et al*., 2003).

Both gases can be released into the atmosphere by gas diffusion at the soil surface and by ebullition in the case of flooded areas. Plants, moreover, can contribute to ecosystem N_2_O and CH_4_ exchange by: (1) taking up both gases from the soil via the root system and transporting them into the atmosphere through the transpiration stream or aerenchyma system and enlarged intercellular spaces (Rusch & Rennenberg, 1998; Machacova *et al*., 2013); (2) producing N_2_O and CH_4_ directly in plant tissues (Smart & Bloom, 2001; Keppler *et al*., 2006); (3) consuming N_2_O and CH_4_ from the atmosphere by a nonspecified mechanism (Sundqvist *et al*., 2012; Machacova *et al*., 2016b, 2017, 2019); and (4) altering the N and C turnover processes in adjacent soil (Menyailo & Hungate, 2005; Yu & Chen, 2009). Moreover, cryptogamic stem covers (i.e. photoautotrophic organisms associated with tree bark, such as lichens, liverworts, mosses or ferns) also might contribute to N_2_O and CH_4_ exchange of trees and forest ecosystems (Lenhart *et al*., 2015; Machacova *et al*., 2017). Especially (but not solely) in tropical rain forests, cryptogams often grow on as much as 100% of the tree bark surface and all the way up to the crowns. These organisms remain overlooked as potential players in the trace gas exchange of forests and trees, even though they are present in the majority of tree stem chamber measurements and can, therefore, contribute to the gas exchange between the bark’s surface and the chamber headspace. In summary, the net exchange of trace gases at the soil–plant–atmosphere interfaces results from a balance of simultaneously ongoing processes of gas production and consumption, gas transport within the relevant system, gas emission into the atmosphere, and gas uptake from the atmosphere, all of which together determine whether the forest compartment will be a source or sink of CH_4_ and N_2_O (Barba *et al*., 2019).

Recent research in various climatic zones has revealed that not only soils and herbaceous plants but also woody plants can be significant sources of N_2_O and CH_4_ to the atmosphere (Machacova *et al*., 2013, 2016a,b, 2019; Pangala *et al*., 2013, 2017; Maier *et al*., 2018; Welch *et al*., 2018; Schindler *et al*., 2020). The trace gas exchange capacity of trees and their contributions to ecosystem N_2_O and CH_4_ exchange seem, however, to vary considerably among tree individuals, tree species, forest ecosystem types and climatic zones, and to depend on many aspects, such as soil and site parameters, tree size, age and health conditions, environmental conditions and seasonal dynamics (Barba *et al*., 2019; Covey & Megonigal, 2019; Machacova *et al*., 2019). Even though the interactions between soil, vegetation and atmosphere exert a crucial role in controlling the ecosystem budget of N_2_O and CH_4_, our current – still limited –knowledge on tree exchange of CH_4_, and especially of N_2_O, does not allow us to clearly identify common characteristics, processes, pathways, and mechanisms of N_2_O and CH_4_ exchange in the soil–tree–atmosphere continuum, and to constrain the magnitudes and patterns of N_2_O and CH_4_ emissions.

Wetlands and floodplains are the largest natural sources of atmospheric CH_4_ in the tropics (Saunois *et al*., 2016). To date, calculations of N_2_O and CH_4_ fluxes between wetlands and the atmosphere have been based mostly upon GHG exchange at the soil–atmosphere interface only, thus excluding the exchange activity of such other ecosystem compartments as trees and other vegetation. This approach can lead to underestimating the ecosystem fluxes (Barba *et al*., 2019). In general, trees growing in wetlands and floodplain forests seem to be stronger emitters of CH_4_ than trees in upland forests (Pangala *et al*., 2013, 2017; Machacova *et al*., 2016a,b; Covey & Megonigal, 2019; Jeffrey *et al*., 2019; Sjögersten *et al*., 2020), whereas trees in riparian forests show emission values in between (Schindler *et al*., 2020). The giants for their CH_4_ emission potential are angiosperms in tropical rain forests of the Amazon basin, with CH_4_ emissions two to three orders of magnitude greater than those of trees growing in other tropical and temperate floodplain forests (Pangala *et al*., 2017; Covey & Megonigal, 2019). Pangala *et al*. (2017) showed that these trees adapted to high soil water level are responsible for as much as half of the CH_4_ emissions from the Amazon floodplain, which is the largest natural CH_4_ source in the tropics.

With the exception of detailed, in‐depth studies ongoing in the Amazon basin, which are today often used in estimating the overall CH_4_ exchange of tropical forests, the widely distributed rain forests in other tropical continental and insular areas of Africa and Asia remain understudied. Likewise, mangroves and tropical forests on upland soils also are wholly understudied in relation to the CH_4_ exchange of their woody vegetation (Pangala *et al*., 2013; Welch *et al*., 2018; Jeffrey *et al*., 2019; Sjögersten *et al*., 2020). Moreover, the N_2_O exchange of mature trees growing under their natural field conditions is rarely investigated world‐wide (Díaz‐Pinés *et al*., 2016; Machacova *et al*., 2017, 2019; Wen *et al*., 2017), and information for tropical regions, including tropical rain forests, is scarce (Welch *et al*., 2018). Without ecologically relevant studies of tree and ecosystem exchange of CH_4_ and N_2_O covering the broad mosaic of tropical forest ecosystems, it is more than challenging to understand the role of woody plants in the GHG balance of tropical regions, to correctly estimate the tropical forest CH_4_ and N_2_O budgets, and to predict their future development in relation to global climate change.

Accordingly, the objective of our case study was to quantify the N_2_O, CH_4_ and, additionally, carbon dioxide (CO_2_, an indicator of physiological activity) exchange of dominant tree species in a tropical lowland rain forest on volcanic Réunion Island (southwestern Indian Ocean). We studied trace gas fluxes from two trees endemic for Réunion and Mauritius islands (*Doratoxylon apetalum*, *Antirhea borbonica*) and four regional endemics of Madagascar, Mauritius and Réunion (*Syzygium borbonicum*, *Homalium paniculatum*, *Mimusops balata* and *Labourdonnaisia calophylloides*). These measurements were accompanied by the investigation of GHG exchange from adjacent soil and volcanic surfaces (basaltic lava flows) and from widespread cryptogamic (bryophytic) stem covers (*Pyrrhobryum spiniforme*, *Leucoloma capillifolium*). The volcanic Réunion Island belongs to one of 36 world hotspots of biodiversity with high level of endemism (Kreft *et al*., 2008), and its National Park, covering 42% of the island, provides an exceptional setup for scientific experimentations in primary vegetation along a major elevational gradient (3069 m above sea level (asl), Piton des Neiges) and with proximity to a regularly active volcano (2632 m asl, Piton de La Fournaise).

Still relatively young, the studied forest has developed on a 400‐yr‐old basaltic lava flow. Because young lava flows lack nutrients and organic matter, the microorganisms colonizing these newly created ecosystems fix not only N_2_, CO_2_ and ammonium (NH_4_^+^), but also other trace gases from the atmosphere, such as carbon monoxide (CO), hydrogen (H_2_) and CH_4_, and drive the sequestration of N, C and other nutrients needed for further ecosystem development (King, 2003; Gomez‐Alvarez *et al*., 2007; Byloos *et al*., 2018). The further ecological succession is connected to the accumulation of nutrients and leads to the gradual development of various plant communities starting with algae, mosses, lichens and ferns, followed by woody shrubs, shrubs growing to trees and later mature trees, forming a dense canopy forest *c*. 400 yr after the destruction of all past vegetation by new lava substrate formation (Potgieter *et al*., 2014). This development is accompanied by the formation of a soil layer, with associated changes in soil properties and microbial community. In the case of the studied area, an unique native lowland rain forest has developed (Kirman *et al*., 2007).

Even though the island has a tropical climate with high annual precipitation (> 4000 mm), the studied forest is atypical compared to well‐studied Amazon basin forests. It is specific for its porous volcanic bedrocks with the presence of lava holes and tubes, thin and irregular soil layer with weak water holding capacity, and steep slopes. It hosts high biodiversity, including mostly tree species endemic at the archipelago or regional levels. The aforementioned characteristics have resulted in an unique and atypical tropical rain forest ecosystem without standing water even under heavy rains. Therefore, we aimed to investigate: (1) whether – and, if so, to what extent – the tree stems growing on a lava flow exchange CH_4_ and N_2_O with the atmosphere; (2) how the tree fluxes contribute to the forest GHG exchange; and (3) whether the tropical rain forest is a source or sink for CH_4_ and N_2_O at the beginning of the rainy season.

## Materials and Methods

### Site description and study design

The experiment was conducted within the Mare Longue Nature Reserve (lat. −21°21′28.2024″N, long. 55°44″37.554”E), situated in the southeast of Réunion Island, a tropical volcanic island located in the southwestern Indian Ocean. The studied mixed forest is a tropical lowland rain forest characterized by endemic vegetation typical for the volcanic islands in the Mascarene Archipelago. The studied forest site (size 1 ha, established in 1999 by the University of Réunion Island) is located on sloping terrain at 180–200 m asl and situated on a pahoehoe basaltic lava flow *c*. 400 yr old (Kirman *et al*., 2007). The soil cover is irregular and thin, and consists mostly of organic matter, parent‐rock fragments and iron oxyhydroxides (Kirman, 2003; Meunier *et al*., 2010). The thin A horizon (average 1 cm depth) is accompanied by thin and irregularly occurring eluvial deposits (weathered surface of the basaltic lava bedrocks; i.e. the C horizon). The chemical composition of the basalt (composed of feldspar (anorthite), olivine (forsterite) and pyroxene (augite)) and of the soil in the studied forest site can be found in Meunier *et al*. (2010). In the experimental plots, we distinguished soil‐covered lava flow spots (further referred to as ‘soil’) from bare lava flow surfaces without soil coverage (further referred to as ‘basaltic lava flows’ or ‘volcanic rocks’). As a consequence of the roughness of the soil surface, the plant roots colonize deep volumes of lava bedrocks (Meunier *et al*., 2010). The aboveground biomass and litterfall of the studied forest were measured in the previous study of Kirman *et al*. (2007). The total standing biomass (535 t ha^–1^) and annual litterfall (7.6 t ha^–1^), as well the chemical composition of the biomass (stored major elements), are similar to other tropical rain forests (Kirman *et al*., 2007; Meunier *et al*., 2010).

The long‐term mean annual precipitation of the southeast of Réunion Island is 4256 mm; the mean minimum and maximum temperatures are 19.9 and 26.4°C, respectively (data from Le Baril Météo‐France station, 1981–2010). The study period (8 October–7 November 2018) was characterized by mean daily air and soil temperature (50 cm soil depth) of 20.6 ± 2.0°C and 19.3 ± 0.5°C (mean ± SD), respectively, and mean daily relative humidity of 97.4% ± 7.1%. All daily parameters were measured in the studied forest site using an RHTemp1000IS relative humidity and temperature data logger (MadgeTech, Warner, NH, USA), and Soil Matric Potential Sensors 253‐L and 257‐L (Campbell Scientific, Logan, UT, USA). The measurement period was a time of transition between a cool and dry season (May–October) and a warm and humid season (November–April).

Within the studied forest site area of 1 ha, we randomly selected 17 experimental plots (each 100 m^2^) out of 100 available experimental plots. The exchange of nitrous oxide (N_2_O), methane (CH_4_) and carbon dioxide (CO_2_) from stems was studied on six dominant tree species (in total 24 mature trees representatively selected within these plots): *Syzygium borbonicum* J. Guého et A.J. Scott (*n* = 5); *Doratoxylon apetalum* (Poir.) Radlk. var. *apetalum* (*n* = 5); *Antirhea borbonica* J.F. Gmel (*n* = 5); *Homalium paniculatum* (Lam.) Benth. (*n* = 3); *Mimusops balata* (Aubl.) C.F. Gaertn. (*n* = 3); and *Labourdonnaisia calophylloides* Bojer (*n* = 3). The biometric parameters of the measured trees can be found in Table 1. Moreover, cryptogamic (bryophytic) stem covers (i.e. photoautotrophic organisms growing on tree bark) typical for the selected trees (*Pyrrhobryum spiniforme* (Hedw.) Mitt., *Leucoloma capillifolium* Renauld; *n* = 4) were collected for further trace gas flux measurements under laboratory conditions. The exchange of greenhouse gases (GHG) from the adjacent soil was measured close to each selected individual tree (total *n* = 24 soil positions). Finally, basaltic lava flows as volcanic surfaces without soil cover (*n* = 8) were studied for their N_2_O, CH_4_ and CO_2_ exchange potential.

**Table 1:** Biometric parameters of studied trees and forest stand characteristics (mean ± SD).

Tree	DBH (m)	Tree height* (m)	Stem surface area (m^2^)	Tree density** (trees ha^−1^)
Syzbor	0.33 ± 0.08	18.8 ± 4.4	10.2 ± 5.0	30
Dorape	0.25 ± 0.05	13.9 ± 2.7	5.5 ± 2.2	91
Antbor	0.16 ± 0.02	13.1 ± 1.9	3.4 ± 1.0	86
Hompan	0.43 ± 0.23	16.0 ± 7.8	12.8 ± 12.6	42
Mimbal	0.42 ± 0.17	23.7 ± 9.4	17.3 ± 14.1	65
Labcal	0.54 ± 0.21	28.5 ± 10.6	26.2 ± 19.3	58

Syzbor, *Syzygium borbonicum* (*n* = 5); Dorape, *Doratoxylon apetalum* (*n* = 5); Antbor, *Antirhea borbonica* (*n* = 5); Hompan, *Homalium paniculatum* (*n* = 3); Mimbal, *Mimusops balata* (*n* = 3); Labcal, *Labourdonnaisia calophylloides* (*n* = 3); DBH, stem diameter at breast height.

* Tree height of studied trees was estimated using allometric relationships for Mare Longue Nature Reserve kindly provided by Olivier Flores (University of Réunion Island).

** Tree density is calculated for each tree species separately and for trees with DBH > 0.10 m to include only trees relevant for flux measurements. The tree density of all six tree species studied is 372 trees ha^−1^. The overall forest density, including all *c*. 80 tree species present, is estimated to be 2150 trees ha^−1^ (Kirman *et al*., 2007).

The measurements of N_2_O, CH_4_ and CO_2_ fluxes from stems and soil were made in pairs, in the sense that the stem gas flux measurement for an individual tree directly followed the gas flux measurement of the adjacent soil to ensure measurements under similar environmental and climatic conditions. The measurements on cryptogams and basaltic lava flows were carried out in blocks. All fluxes were determined between 09:00 h and 18:00 h. One measurement set from all selected trees, cryptogams, soils and basaltic lava flows required *c*. 1.5–2 wk. All of the forest components were measured twice.

### Gas sampling from stems, soil and basaltic lava flows

**Fig. 1:**
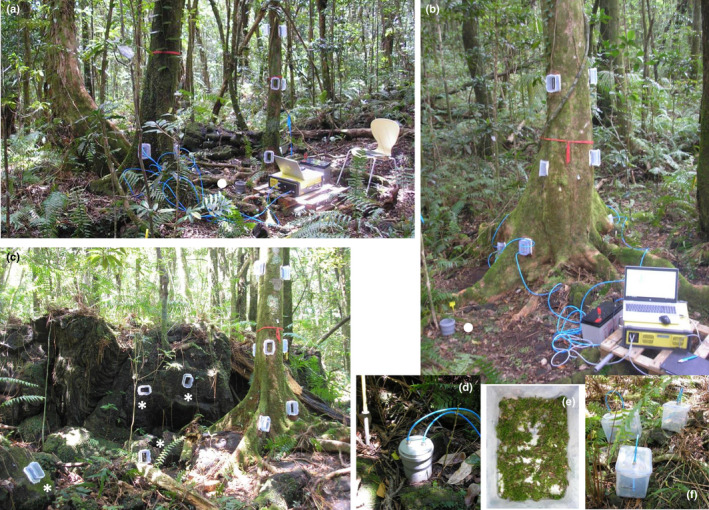
View of study set‐up and chamber systems used to determine greenhouse gas fluxes in the lowland tropical rain forest on volcanic Réunion Island. Overall view of the site: stem chambers installed in the vertical profile of tree stems (a–c), soil chambers in the vicinity of studied trees (a, b; marked with white circle; and d), volcanic rock chambers fixed on basaltic lava flow (c, marked with white asterisk), cryptogamic stem covers in incubation chambers (e, f), and portable greenhouse gas analyzer used for gas concentration measurements (a, b).

Stem fluxes of N_2_O, CH_4_ and CO_2_ were measured at the bottom part of the stems (*c*. 0.4 m aboveground) for all selected tree species and tree individuals. The vertical profile of the stem fluxes (measurements at three stem heights of *c*. 0.4, 1.1 and 1.8 m aboveground; Fig. 1a–c) was studied in three individual trees of each of the following three species: *S. borbonicum*, *D. apetalum* and *A. borbonica*. The stem fluxes were measured using static stem chamber systems installed at the beginning of the measurement campaign. The chambers consisted of transparent plastic containers with removable airtight lids (Lock & Lock, Seoul, South Korea) and a neoprene sealing frame. They were gas‐tightly affixed to the carefully smoothed bark surface *c*. 2 wk before measurements. Control measurements were performed to ensure that the observed fluxes did not originate from the chamber materials used. On each tree other than *S. borbonicum* individuals, two chambers were installed at one stem height on opposite sides of the stem and interconnected with polyurethane tubes into a single flow‐through chamber system. The total enclosed stem area was 0.0108 m^2^ and total internal system volume was 0.0021 m^3^. (For more details, see Machacova *et al*., 2015, 2017, 2019.) Owing to the large stem diameter of *S. borbonicum*, three chambers (total area 0.0162 m^2^, total internal volume 0.0028 m^3^) were installed at one stem height in the same manner on those trees. Gas‐tightness of all the chambers was tested regularly using CO_2_ signal and a portable gas analyzer (see the ‘Flux measurements and calculations’ section below).

Soil N_2_O, CH_4_ and CO_2_ fluxes were measured using static soil chamber systems (Fig. 1b,d). The soil collars were installed in the vicinity of the investigated trees at the beginning of the research stay to reduce soil disturbances. As a result of the shallow soil layer on the lava flow, the bottom part of the collars was imbedded to a maximum 2.5 cm deep into the soil. The soil chambers were made of sewage pipes (total enclosed soil area 0.0083 m^2^, total internal system volume *c*. 0.0015 m^3^ depending on soil depth). During measurements, the soil chambers were closed by a lid and sealed with water between the body of the chamber and the lid (Machacova *et al*., 2013).

The fluxes of N_2_O, CH_4_ and CO_2_ from basaltic lava flows were determined using the stem chambers as described above but installed directly onto the volcanic surfaces (Fig. 1c). One flow‐through chamber system consisted of one chamber (total enclosed rock area 0.0054 m^2^, total internal system volume 0.0013 m^3^) connected to a gas analyzer (see the ‘Flux measurements and calculations’ section below).

### Collection and incubation of cryptogamic stem covers

*Pyrrhobryum spiniforme* and *Leucoloma capillifolium* mosses were collected from the bark of *S. borbonicum* trees. To avoid disruption to the bark microcosm within the stem chambers, the cryptogams were collected from bark outside the measurement chambers. After collection, fresh samples were placed into plastic gas‐tight incubation chamber (0.0039 m^3^ volume; Lock & Lock; Fig. 1e,f) connected to a gas analyzer and incubated in the laboratory under dark conditions and low light intensity of 10–15 µmol m^−2^ s^−1^ corresponding to the low light conditions of forest understories.

The stem, soil, lava flow and incubation chamber systems were left open during the time between the individual measurements.

### Flux measurements and calculations

For GHG flux measurements, the measurement chambers were closed with lids, and the concentration changes of N_2_O, CH_4_ and CO_2_ in chamber headspace were measured using a Gasmet DX‐4015 portable Fourier transform infrared (FTIR) gas analyzer (Gasmet Technologies Oy, Vantaa, Finland; Warlo *et al*., 2018). A single measurement run of stem, soil and lava flow gas fluxes lasted *c*. 45 min. The measurement time for cryptogams was 180–240 min, depending on flux rates. The internal pump (0.002 m^3^ min^−1^) of the analyzer ensured mixing of air inside the chamber systems. Every morning, zero‐point calibration of the analyzer was made using N_2_ (99.9992% purity).

The gas exchanges of stems, soil, lava flows and cryptogams were quantified based upon the linear changes in N_2_O, CH_4_ and CO_2_ concentrations in the chamber headspace over time (for examples, see Supporting Information Fig. S1; for equations used, see Machacova *et al*., 2016a). Decrease of gas concentration over time indicated gas uptake (i.e. negative flux), increase of gas concentration indicated gas emission (i.e. positive flux). The fluxes from tree stems and soil were further roughly scaled up to the ecosystem level based on tree and forest characteristics (stem diameter at breast height (DBH), tree height, tree density for individual tree species with trees of DBH > 0.10 m; Table 1; Machacova *et al*., 2016a). The scaling up of trace gas exchange of volcanic surfaces of lava flows was not possible because the exact extent of volcanic bedrock surfaces without soil cover within the studied forest was unknown. The contribution of cryptogams to the stem GHG fluxes was estimated based on upscaling of the fluxes related to g DW (dry weight) to the unit of stem surface area (Machacova *et al*., 2017).

### Statistics

The flux data were checked for normal distribution (Shapiro–Wilk test) and equality of variances in the different subpopulations. Student's *t*‐test and one‐way ANOVA for multiple comparisons were applied for normally distributed data. The nonparametric Mann‒Whitney rank‐sum test and Kruskal–Wallis one‐way ANOVA on ranks for multiple comparisons were applied for non‐normally distributed data or data with unequal variances. The exact applied tests and *n* values for statistical analyses are stated in the figure legends and in the ‘Site description and study design’ section above, respectively. The study was performed in 17 randomly selected experimental plots within the studied 1 ha forest site, and the studied trees together with the soil positions and basaltic lava flow surfaces were representatively chosen within these plots. Statistical significance for all tests was defined as *P* < 0.05. The statistics were run using sigmaplot 11.0 (Systat Software, San Jose, CA, USA).

## Results

### Methane exchange of forest compartments

**Fig. 2:**
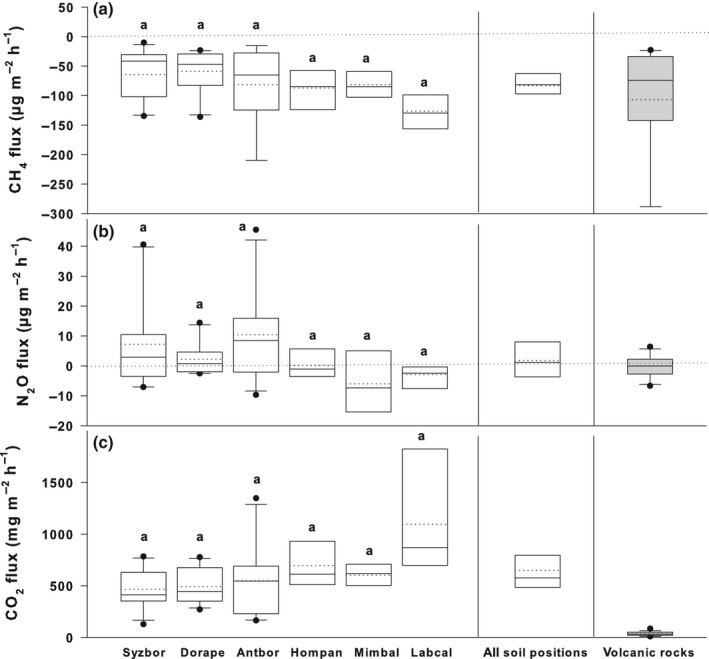
Fluxes of methane (CH_4_) (a), nitrous oxide (N_2_O) (b), and carbon dioxide (CO_2_) (c) from adjacent soil measured close to each studied tree and from volcanic rock surfaces of basaltic lava flows. The fluxes are expressed as medians (solid lines) and means (dashed lines) of measurements from soil positions in the vicinity of trees of each individual tree species (Syzbor, *Syzygium borbonicum* (*n* = 5); Dorape, *Doratoxylon apetalum* (*n* = 5); Antbor, *Antirhea borbonica* (*n* = 5); Hompan, *Homalium paniculatum* (*n* = 3); Mimbal, *Mimusops balata* (*n* = 3); Labcal, *Labourdonnaisia calophylloides* (*n* = 3)), from all studied soil positions (‘All soil positions’, *n* = 24), and from volcanic rocks (*n* = 8). The forest floor in the studied forest is approximately equally covered with soil and volcanic surfaces without soil cover (proportion roughly 50 : 50). Fluxes are expressed per m^2^of soil and volcanic rock area. Positive fluxes indicate trace gas emission; and negative fluxes trace gas uptake. The box boundaries mark the 25^th^ and 75^th^ percentiles and whiskers the 10^th^ and 90^th^ percentiles. Dots mark outliers. As indicated by the letter ‘a’ above each bar, there were no statistically significant differences among fluxes in adjacent soil of individual tree species at *P* < 0.05. One‐way ANOVA was applied for CH_4_ fluxes and Kruskal–Wallis one‐way ANOVA on ranks was used for N_2_O and CO_2_ fluxes.

The soil of the tropical lowland rain forest consistently consumed CH_4_ from the atmosphere (–83.3 ± 9.8 µg‐CH_4_ m^−2^ h^−1^ (soil area), mean ± SE) at the beginning of the rainy season. Uniform CH_4_ consumption was observed at all subsites with low, nonsignificant spatial flux heterogeneity (Fig. 2a). The volcanic surfaces of basaltic lava flows also showed consistent uptake of CH_4_ from the atmosphere (−107 ± 21.6 µg‐CH_4_ m^−2^ h^−1^ (rock area), Fig. 2a). As the similar consumption rates of irregularly spread soil layers and of volcanic rocks are expressed per ‘ground’ area, and both surface types seem to be equally distributed in the studied forest, we can confidently say that the detected CH_4_ consumption of both forest compartments equally and significantly contributes to CH_4_ exchange of the forest ground area (Fig. 2a). The forest floor CH_4_ fluxes can therefore be estimated using 50:50 proportion of soil and volcanic rock coverage.

**Fig. 3:**
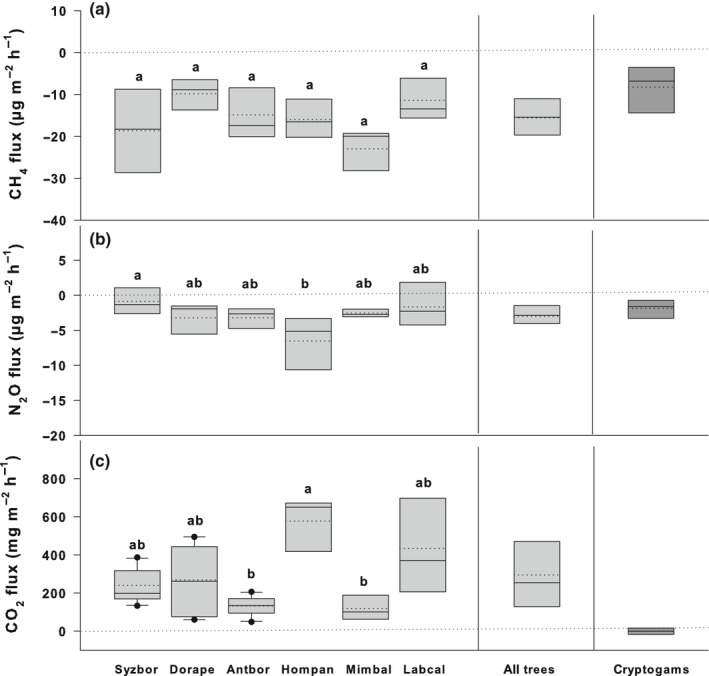
Fluxes of methane (CH_4_) (a), nitrous oxide (N_2_O) (b), and carbon dioxide (CO_2_) (c) from tree stems and cryptogamic stem covers. The fluxes are expressed as medians (solid lines) and means (dashed lines) of measurements from stems of six individual tree species (Syzbor, *Syzygium borbonicum* (*n* = 5); Dorape, *Doratoxylon apetalum* (*n* = 5); Antbor, *Antirhea borbonica* (*n* = 5); Hompan, *Homalium paniculatum* (*n* = 3); Mimbal, *Mimusops balata* (*n* = 3); Labcal, *Labourdonnaisia calophylloides* (*n* = 3)), from all studied trees and tree species (‘All trees’, *n* = 24), and from cryptogams (*Pyrrhobryum spiniforme*, *Leucoloma capillifolium*; *n* = 4). The trace gas exchange of cryptogams is presented as mean and median of all measurements under low light and dark conditions, because CH_4_ and N_2_O fluxes did not differ in relation to light conditions. The mean and median fluxes of CO_2_ in cryptogams include both CO_2_ emission and uptake measured under dark and light conditions, resulting in low gas exchange (more details in manuscript text). All fluxes, including fluxes from cryptogams, are expressed per m^2^ of stem area. Positive fluxes indicate trace gas emission and negative fluxes trace gas uptake. The box boundaries mark the 25^th^ and 75^th^ percentiles and whiskers the 10^th^ and 90^th^ percentiles. Dots mark outliers. Statistically significant differences among fluxes in individual tree species at *P* < 0.05 are indicated by different letters above bars. One‐way ANOVA was applied for CH_4_ and N_2_O fluxes and Kruskal–Wallis one‐way ANOVA on ranks was used for CO_2_ fluxes.

**Fig. 4:**
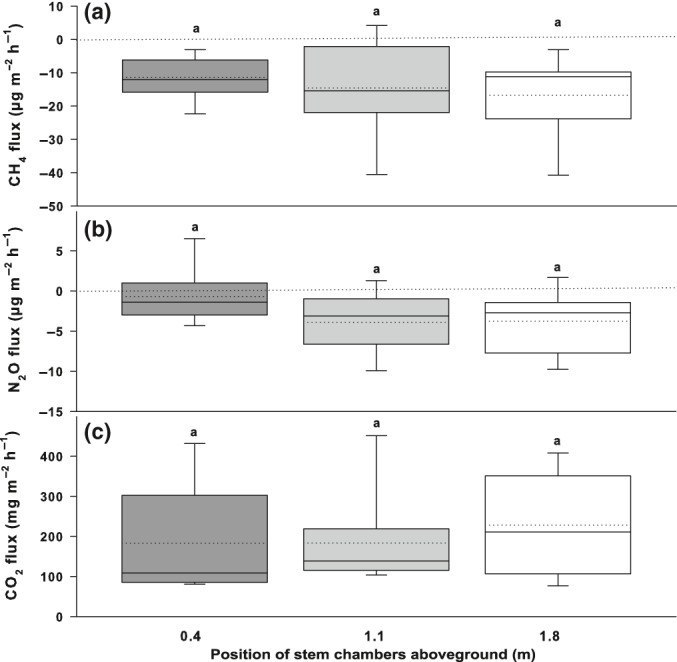
Fluxes of methane (CH_4_) (a), nitrous oxide (N_2_O) (b), and carbon dioxide (CO_2_) (c) from tree stem vertical profiles. The fluxes are expressed as medians (solid lines) and means (dashed lines) of measurements from trees of three tree species: *Syzygium borbonicum* (*n* = 3), *Doratoxylon apetalum* (*n* = 3) and *Antirhea borbonica* (*n* = 3). The measurements were performed at three stem heights of *c*. 0.4, 1.1 and 1.8 m aboveground. All fluxes are expressed per m^2^ of stem area. Positive fluxes indicate trace gas emission and negative fluxes trace gas uptake. The box boundaries mark the 25^th^ and 75^th^ percentiles and whiskers the 10^th^ and 90^th^ percentiles. As indicated by the letter ‘a’ above each bar, there were no statistically significant differences in fluxes among stem heights at *P* < 0.05. One‐way ANOVA was applied for CH_4_ and N_2_O fluxes and Kruskal–Wallis one‐way ANOVA on ranks was used for CO_2_ fluxes.

**Fig. 5:**
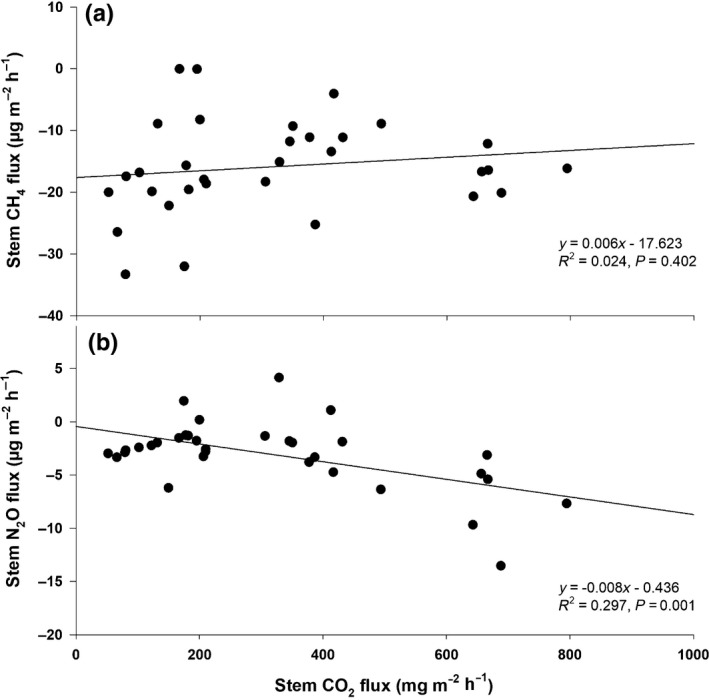
Relationships between methane (CH_4_) vs carbon dioxide (CO_2_) stem fluxes (a) and nitrous oxide (N_2_O) vs CO_2_ stem fluxes (b). All six tree species (24 trees in total) are included. All fluxes are expressed per m^2^of stem area. Positive flux values indicate gas emission and negative values indicate gas uptake.

All mature studied tree stems were net sinks of CH_4_ from the atmosphere (−15.6 ± 2.0 µg‐CH_4_ m^−2^ h^−1^ (stem area)). The CH_4_ consumption was uniform across the various tree species and did not show significant species‐specific variability (Fig. 3a). Moreover, no significant changes in stem CH_4_ consumption were observed with respect to stem height aboveground (Fig. 4a). The uptake of CH_4_ by tree stems was not closely connected to stem CO_2_ efflux (Fig. 5a). To better understand the mechanisms behind the observed CH_4_ uptake by trees, the GHG exchange of widespread cryptogamic stem cover was measured. We estimated how much CH_4_ is taken up by the active cryptogams per stem area unit when fully covered with these organisms. As a result, cryptogams were identified as net sinks of CH_4_ (–8.3 ± 3.0 µg‐CH_4_ m^−2^ h^−1^ (stem area); −0.047 ± 0.016 µg g^−1^ h^−1^(DW); Fig. 3a). All main forest compartments studied therefore consumed CH_4_ from the atmosphere.

**Fig. 6:**
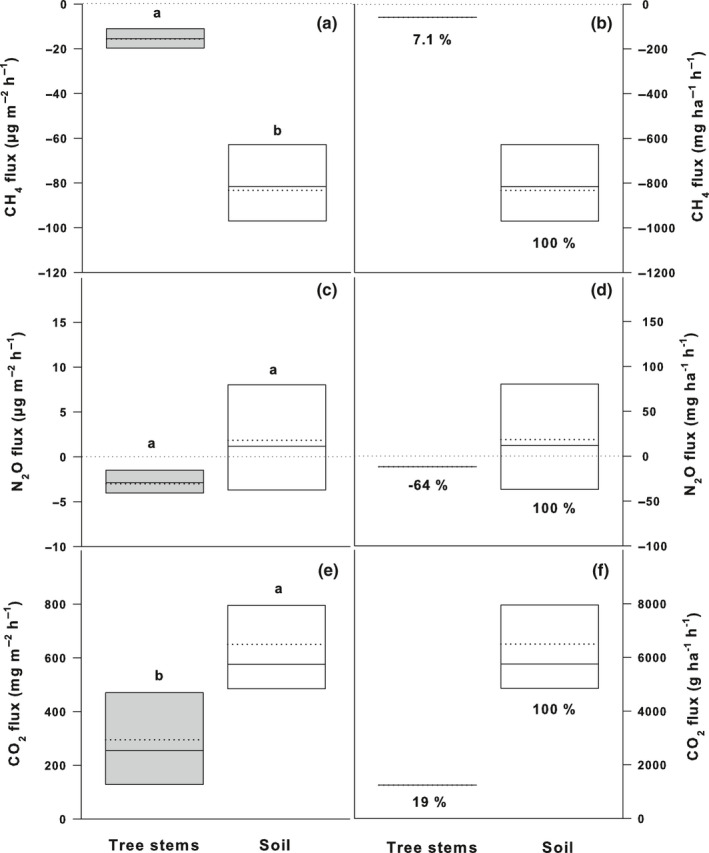
Fluxes of methane (CH_4_) (a, b), nitrous oxide (N_2_O) (c, d) and carbon dioxide (CO_2_) (e, f) from tree stems and adjacent soil expressed per stem or soil surface area unit (a, c, e) and scaled up to unit ground area of the tropical rain forest (b, d, f). The fluxes are expressed as medians (solid lines) and means (dashed lines) of measurements from all studied trees of six tree species (*n* = 24) and all studied soil positions (*n* = 24). Positive fluxes indicate trace gas emission; negative fluxes trace gas uptake. The box boundaries mark the 25^th^ and 75^th^ percentiles and whiskers the 10^th^ and 90^th^ percentiles. Statistically significant differences in fluxes at tree stem and soil level at *P* < 0.05 are indicated by different letters above the bars. Mann–Whitney rank‐sum test was applied for the gas flux pairs. The contributions of stem fluxes of six tree species to the soil fluxes (equal to 100%) are expressed as percentages of the soil flux.

In order to estimate the contribution of the tree stems to the tropical rain forest CH_4_ exchange, the detected stem and soil consumption rates were scaled up to ecosystem level (Fig. 6a,b). The six tree species studied (joint tree density of *c*. 370 trees ha^−1^) consumed in total –59.0 ± 17.9 mg‐CH_4_ ha^−1^ h^−1^ (ground area) (i.e. the sum of mean tree species consumption rates and SEs) and contributed 7.1% to the soil CH_4_ uptake (–833.1 ± 97.5 mg‐CH_4_ ha^−1^ h^−1^; Fig. 6b). As there are > 80 tree species present in the studied forest (total tree density of *c*. 2150 trees ha^−1^; Kirman *et al*., 2007), we estimate that the overall contribution of all tree stems present to the soil CH_4_ uptake might be 41% when assuming similar CH_4_ uptake potential by all tree species present. This tropical rain forest with its > 80 tree species seems to be a strong sink for CH_4_ at the beginning of the rainy season (−1176 mg‐CH_4_ ha^−1^ h^−1^).

### Nitrous oxide exchange of forest compartments

The soil was on average a weak source of N_2_O (1.8 ± 2.5 µg‐N_2_O m^−2^ h^−1^; Fig. 2b). The detected exchange potential of volcanic surfaces of basaltic lava flows was negligible (0.024 ± 0.871 µg‐N_2_O m^−2^ h^−1^). The exchange of soil and volcanic surfaces showed high spatial heterogeneity without any clear trend, including both N_2_O consumption and emission. Moreover, the majority of the exchange rates were very low.

In contrast, clear uptake of N_2_O by tree stems was observed. Thus, these are net sinks of N_2_O from the atmosphere (–3.0 ± 0.8 µg‐N_2_O m^−2^ h^−1^; Fig. 3b). The lowest N_2_O consumption was detected for *Syzygium borbonicum* and the highest for *Homalium paniculatum*. The uptake of N_2_O by tree stems was negatively correlated with stem CO_2_ efflux (Fig. 5b). Similar to the situation for CH_4_, no significant changes in stem N_2_O exchange were observed within the vertical stem profile (Fig. 4b). The cryptogams were a clear sink not only of CH_4_, but also of N_2_O (−1.9 ± 0.7 µg‐N_2_O m^−2^ h^−1^ (stem area); −0.011 ± 0.004 µg g^−1^ h^−1^ (DW); Fig. 3b). Our test measurements confirmed that the observed cryptogamic N_2_O and CH_4_ uptake could not be explained by gas dilution in air humidity within the incubation chambers (Machacova *et al*., 2017).

The scaled‐up rates of N_2_O consumption by tree stems of the six dominant tree species studied (−11.9 ± 6.3 mg‐N_2_O ha^−1^ h^−1^) represented −64% of the soil N_2_O emissions (18.4 ± 25.2 mg‐N_2_O ha^−1^ h^−1^; Fig. 6c,d), thus decreasing the weak source strength of the soil. (Please note that the usage of the negative percentage in case of N_2_O is due to comparison of bidirectional fluxes – negative fluxes (i.e. N_2_O uptake by stems) with positive fluxes (i.e. N_2_O emission by soil; equal to 100%). In the case of CH_4_, the percentage contribution of the stem fluxes to the soil fluxes is positive, as both – tree stems and soil – are sinks for CH_4_.) Under the assumption of all 80 tree species in the forest being N_2_O consumers at similar uptake rates, however, we expect that the overall N_2_O uptake potential of all trees (–68.9 mg‐N_2_O ha^−1^ h^−1^) might represent even –374% of the soil N_2_O exchange, thus turning the tropical rain forest into a sink of N_2_O (–50.45 mg‐N_2_O ha^−1^ h^−1^).

### Carbon dioxide exchange of forest compartments

The measured CO_2_ exchange of forest compartments is an ancillary parameter (i.e. an indicator of physiological activity) helping to understand the CH_4_ and N_2_O exchange in the soil–tree–ecosystem–atmosphere continuum. The CO_2_ emissions from soil substantially exceeded those from volcanic surfaces of lava flows (650 ± 95 and 35.5 ± 4.7 mg‐CO_2_ m^−2^ h^−1^, respectively; Fig. 2c). Tree stems emitted 295 ± 73 mg‐CO_2_ m^−2^ h^−1^, with *Homalium paniculatum* being the strongest emitter (Fig. 3c). The stem CO_2_ efflux was uniform across the vertical stem profile (Fig. 4c). As expected, photoautotrophic cryptogamic stem covers consumed CO_2_ under light conditions (−15.6 ± 2.5 mg‐CO_2_ m^−2^ h^−1^ (stem area); –0.094 ± 0.027 mg g^−1^ h^−1^(DW)) and emitted CO_2_ under dark conditions (14.8 ± 2.9 mg‐CO_2_ m^−2^ h^−1^ (stem area); 0.087 ± 0.010 mg g^−1^ h^−1^(DW); NB Fig. 3c shows total mean and median values from both incubation conditions).

## Discussion

### All studied forest compartments are net methane sinks

#### Soil and basaltic lava flows

Soil (i.e. soil on soil‐covered lava flow spots) and basaltic lava flows (i.e. bare lava flow surfaces without soil coverage) consistently consumed methane (CH_4_) from the atmosphere, even though the measurements were done at the start of the rainy season. The forest floor is covered by irregular and thin soil (Kirman *et al*., 2007), likely preventing the formation and preservation of anaerobic soil conditions for methanogenic CH_4_ production and thus supporting methanotrophic CH_4_ oxidation (Smith *et al*., 2003). The steep slope further promotes rapid rainwater runoff from the forest into the ocean. Moreover, the high porosity typical for the volcanics forming the underlying rocks of the forests and their typical irregular distribution with the presence of hollows and lava tubes also contributes to rapid rainwater runoff and oxygenation of the whole system.

To the best of our knowledge, the ability of basaltic lava flows to exchange CH_4_ with the atmosphere has never been reported. We speculate that the CH_4_ oxidation occurs in volcanic pores covered with a thin layer of biofilm consisting of a broad spectrum of microbial communities, including (*inter alia*) methanotrophs (e.g. Gomez‐Alvarez *et al*., 2007; Byloos *et al*., 2018). Methanotrophs seem to colonize the newly formed lava already within 40 yr, but the colonization is closely related to the development of plant and microbial communities and local climatic conditions (King & Nanba, 2008). We expect that volcanic surfaces’ low water absorption and retention capacity (Gomez‐Alvarez *et al*., 2007; Byloos *et al*., 2018) promote aerobic conditions and support, *inter alia*, CH_4_‐oxidizing microorganisms leading to the observed CH_4_ uptake by volcanic surfaces.

Soil CH_4_ flux measurements on lava flows are rare. The only study conducted on relatively fresh volcanic deposits detected soil CH_4_ uptake in a tropical rain forest (*c*. 300 yr old) on the islands of Hawaii (King, 2003). The CH_4_ consumption rate in that study (reaching –75 µg‐CH_4_ m^−2^ h^−1^) is in accordance with the findings of our study (–83.3 µg‐CH_4_ m^−2^ h^−1^). The similar CH_4_ consumption potentials of soil and volcanic surfaces were accompanied by soil carbon dioxide (CO_2_) emissions substantially exceeding those from volcanic surfaces (Fig. 2c). By comparison, the volcanic deposits in Hawaii produced twice as much CO_2_ (58.8 mg‐CO_2_ m^−2^ h^−1^; King, 2003). The very low CO_2_ emissions of volcanic surfaces in Réunion might be explained by the low availability of organic carbon (C) and resulting low respiration rates (King, 2003).

#### Tree stems and cryptogamic stem covers

Our study clearly determined all mature tree stems of the dominant tree species to be net sinks of CH_4_ (Fig. 3a). To the best of our knowledge, such consistent consumption of CH_4_ by tree stems constitutes an unique finding, because trees are generally known as CH_4_ sources with high individual variability in CH_4_ emission rates, depending on tree species, climatic zone, forest ecosystem type, environmental and meteorological conditions, and seasonal dynamics (Barba *et al*., 2019; Covey & Megonigal, 2019). In particular, stems of Amazonia trees that are well adapted to inundation are regarded as extraordinarily strong CH_4_ sources (Pangala *et al*., 2017). Trees from other tropical areas seem also to play important roles in forest CH_4_ budgets, even with much lower detected emissions than in Amazonia (Pangala *et al*., 2013; Welch *et al*., 2018; Jeffrey *et al*., 2019; Sjögersten *et al*., 2020). It is expected that CH_4_ emitted from these trees mostly originates from the deeper soil layers, as reported for the only study done on tropical upland soils (Welch *et al*., 2018). In that case, trees were observed to serve as ‘chimneys’ for soil‐produced CH_4_ to reach the atmosphere without being oxidized in well‐aerated soil surface layers.

Only boreal tree species are known to be capable of taking up CH_4_ from the atmosphere by their leaves and stems (Sundqvist *et al*., 2012; Machacova *et al*., 2016b), although the stem uptake potential was detected only in the dormant season and was rather negligible (Machacova *et al*., 2016b). Welch *et al*. (2018) detected irregular CH_4_ uptake by stems of *Simarouba amara* in a tropical upland forest in Panama during the transition from dry to wet season. Those authors assumed a diffusion gradient from the atmosphere via tree stems into the soil with predominant CH_4_ oxidation, which might result in tree stem uptake of trace gases.

Our incubation experiments with the most abundant cryptogams on studied trees consistently revealed that all freshly collected cryptogams (bryophytes) were net CH_4_ sinks independently of light conditions (Fig. 3a). The estimated CH_4_ uptake rates per area unit were of the same order of magnitude as stem uptake rates measured under field conditions (Fig. 3a). Therefore, it seems that the epiphytes are co‐responsible for the observed CH_4_ uptake by tropical trees on Réunion. The only known study on cryptogamic CH_4_ exchange presents various cryptogamic species as small CH_4_ sources (0.28 ng‐CH_4_ g^−1^ h^−1^, Lenhart *et al*., 2015; vs –47 ± 16 ng‐CH_4_ g^−1^ h^−1^, this study). We hypothesize that CH_4_‐oxidising microorganisms (i.e. methanotrophs) can be involved in the CH_4_ consumption observed by trees and cryptogams. Future analyses of wood and cryptogams samples for the *pmoA* gene controlling CH_4_ oxidation could bring more explanation to this topic.

In conclusion, the studied tropical rain forest located on a basaltic lava flow seems to be a strong net sink of CH_4_, with soil and volcanic surfaces predominantly being responsible for the CH_4_ uptake. We dare to say that, owing to the reasons stated above, no elevated water table levels should be expected during the rainy season, and the studied tropical rain forest on lava flow might be a substantial sink for CH_4_ all through the year. The stems of the six tree species studied contributed 7.1% to the forest floor CH_4_ uptake (Fig. 6b). As there are > 80 tree species present with high tree density, we estimate that the overall contribution of all tree stems to the forest floor CH_4_ uptake might be as much as 41% and the overall forest uptake potential can reach to −1176 mg‐CH_4_ ha^−1^ h^−1^. The trees growing in the tropical rain forest on lava flows, therefore, are significant contributors to ecosystem CH_4_ uptake. Theoretically, the contribution can be even greater, as foliage, known from the first available studies as important contributors to ecosystem CH_4_ exchange (Machacova *et al*., 2016a), is not included in this case study.

### Tree stems and cryptogamic stem covers are net nitrous oxide sinks

By contrast with their relationship to CH_4_, soil and basaltic lava flows do not significantly exchange nitrous oxide (N_2_O) with the atmosphere (Fig. 2b). The reason might be nitrogen (N) limitation in the thin soil layer, thus resulting in very limited biomass increment in the studied forest over the past 10 yr (O. Flores, pers. comm.; Bennici, 2015). Moreover, the young lava flow is probably still poor in N‐rich nutrients and organic compounds (King, 2003; Gomez‐Alvarez *et al*., 2007).

Nevertheless, the studied trees were identified as net sinks of N_2_O (Fig. 3b). Their uptake potential was weakly and negatively correlated with stem CO_2_ efflux (Fig. 5b), which means that low N_2_O consumption by tree stems was associated with low stem CO_2_ efflux. The net stem CO_2_ efflux, an indicator of tree physiological activity, is a result of stem respiration, radial CO_2_ diffusion from the transpiration stream and CO_2_ re‐fixation on the stem surface (Aubrey & Teskey, 2009; Hölttä & Kolari, 2009; Bloemen *et al*., 2013). A physiological dependence of N_2_O exchange has been observed in boreal trees, whose seasonality in stem N_2_O release followed the tree physiological activity, particularly processes of CO_2_ uptake and release (Machacova *et al*., 2019). A closer connection between plant N_2_O and CO_2_ fluxes was detected early under a wide range of controlled environmental conditions in species belonging to cryptogamic covers (Lenhart *et al*., 2015; Machacova *et al*., 2017) and Spermatophyta (Machacova *et al*., 2017; Lenhart *et al*., 2019).

Similar to tree stems, cryptogams covering the studied tree stems up to the crowns also were detected as net N_2_O sinks, and their N_2_O uptake rates were of the same order of magnitude (Fig. 3b). These findings are unique, as the majority of studies on mature trees under natural field conditions present trees only as weak N_2_O emitters (Machacova *et al*., 2019). The only known study presenting trees (European beech, temperate mountain forest) as substantial net sinks of N_2_O showed cryptogamic stem covers to be co‐responsible for the observed N_2_O uptake by tree stems (Machacova *et al*., 2017). We hypothesize that the N_2_O, which is taken up by the cryptogams, might be reduced to N_2_ in the final step of the denitrification pathway. Some irregular N_2_O uptake by trees also has been observed by boreal trees (Machacova *et al*., 2019) and tropical upland trees (Welch *et al*., 2018). In general, trees seem to exchange N_2_O (and CH_4_) with the atmosphere in both directions under certain conditions. However, the processes and mechanisms behind and fate of the N_2_O (and CH_4_) taken up by the tree stems and cryptogams are still unknown and warrant further investigation.

The studied tree species contributed −64% to soil N_2_O exchange and, therefore, reduced the weak soil N_2_O emission potential (Fig. 6d). We expect an overall N_2_O uptake potential of all tree species in the forest might represent as much as −374% of the soil exchange, thus turning the tropical rain forest into a strong sink of N_2_O (−50.45 mg‐N_2_O ha^−1^ h^−1^).

### Conclusion and future perspectives

This study shows for the first time that native trees in tropical lowland rain forest growing on a basaltic lava flow can be important sinks of atmospheric CH_4_ and N_2_O and can contribute as much as 41% and −374% to the soil CH_4_ and N_2_O exchange, respectively. Also, cryptogamic stem covers were identified as substantial CH_4_ and N_2_O sinks. This ‘plant’ uptake was accompanied by strong CH_4_ uptake by soil and volcanic surfaces of the basaltic lava flow.

In relation to ecosystem CH_4_ exchange, the lowland rain forests growing on lava flows seem to behave differently from more commonly studied rain forests, not acting as strong CH_4_ sources but rather as significant CH_4_ sinks. More studies in different tropical volcanic regions are needed to understand the trace gas exchange potential and mechanisms of these specific ecosystems.

Because our case study was limited by time, the results need to be extended by examining the seasonal variability in CH_4_ and N_2_O exchange for a larger set of tree species, further including fluxes from leaves, and simultaneous measurements of soil and plant chemical and hydrological properties following the previous work of Meunier *et al*. (2010), and seeking to understand the role of cryptogams and basaltic lava flows in the CH_4_ and N_2_O exchange under different climatic conditions.

Our results highlight the importance of previously unexplored lowland tropical rain forests on basaltic lava flows in CH_4_ and N_2_O uptake and, therefore, can be used to improve national greenhouse gas (GHG) inventories. As the studied lowland tropical rain forest in Mare Longue Nature Reserve falls within the range of tropical rain forests (biogeochemistry, biomass increment, litterfall; Kirman *et al*., 2007; Meunier *et al*., 2010), this study can be justified as a model ecosystem of lowland tropical rain forests. These forests are the most species‐rich ecosystems in the world and are of high importance in regulating C and water cycling in the global climate system. However, they are under the greatest pressures from anthropogenic disturbances (acceleration of deforestation of original lowland tropical rain forests, impacts of global warming and drought events) and start to occur only as isolated forest fragments with decreasing biodiversity (Turner & Corlett, 1996; Corlett, 2011; Corlett & Primack, 2011). Confirming the unequivocal CH_4_ and N_2_O uptake potential by future studies would bolster the argument that the protection of such dynamic ecosystems in volcanic regions around the world should be strengthened not only because of their great biodiversity but also for their contribution to reducing GHGs in the atmosphere.

## Author contributions

KM had the idea for the study; KM and CA designed the study; KM, TA and LB carried out the field measurements and analyzed the data; and KM, CA, TS, ÜM, KS and LB contributed to writing the manuscript.

## Supporting information


**Fig. S1** Examples of CH_4_, N_2_O and CO_2_ concentration changes over time in the headspaces of soil chambers, volcanic rock chambers, tree stem chambers and incubation chambers containing cryptogams.
Please note: Wiley Blackwell are not responsible for the content or functionality of any Supporting Information supplied by the authors. Any queries (other than missing material) should be directed to the *New Phytologist* Central Office.

## Data Availability

The datasets generated and analyzed during this study are available from the corresponding author upon reasonable request.

## References

[nph17002-bib-0001] Aubrey DP , Teskey RO . 2009 Root‐derived CO_2_ efflux via xylem stream rivals soil CO_2_ efflux. New Phytologist 184: 35–40. 10.1111/j.1469-8137.2009.02971.x 19674328

[nph17002-bib-0002] Barba J , Bradford MA , Brewer PE , Bruhn D , Covey K , van Haren J , Megonigal JP , Mikkelsen TN , Pangala SR , Pihlatie M *et al* 2019 Methane emissions from tree stems: a new frontier in the global carbon cycle. New Phytologist 222: 18–28. 10.1111/nph.15582 30394559

[nph17002-bib-0003] Bennici A . 2015 Modélisation de la dynamique d’une forêt tropicale humide insulaire: Mare‐Longue, La Réunion. MSc thesis, Université de la Réunion, Réunion, France.

[nph17002-bib-0004] Bloemen J , McGuire MA , Aubrey DP , Teskey RO , Steppe K . 2013 Transport of root‐respired CO_2_ via the transpiration stream affects aboveground carbon assimilation and CO_2_ efflux in trees. New Phytologist 197: 555–565. 10.1111/j.1469-8137.2012.04366.x 23057485

[nph17002-bib-0005] Byloos B , Monsieurs P , Mysara M , Leys N , Boon N , Van Houdt R . 2018 Characterization of the bacterial communities on recent Icelandic volcanic deposits of different ages. BMC Microbiology 18: 122. 30249184 10.1186/s12866-018-1262-0 PMC6154810

[nph17002-bib-0006] Corlett RT . 2011 Impacts of warming on tropical lowland rainforests. Trends in Ecology and Evolution 26: 606–613. 21803440 10.1016/j.tree.2011.06.015

[nph17002-bib-0007] Corlett RT , Primack RB . 2011 Tropical rain forests: an ecological and biogeographical comparison. Hoboken, NJ, USA: Wiley‐Blackwell.

[nph17002-bib-0008] Covey KR , Megonigal JP . 2019 Methane production and emissions in trees and forests. New Phytologist 222: 35–51. 10.1111/nph.15624 30521089

[nph17002-bib-0009] D’Annunzio R , Lindquist EJ , MacDicken KG , eds. 2017 Global forest land‐use change from 1990 to 2010: an update to a global remote sensing survey of forests. Forest Resource Assessment Working Paper 187. Rome, Italy: Food and Agriculture Organization of the United Nations.

[nph17002-bib-0010] Dalal RC , Allen DE . 2008 Turner review no. 18: Greenhouse gas fluxes from natural ecosystems. Australian Journal of Botany 56: 369–407.

[nph17002-bib-0011] Díaz‐Pinés E , Heras P , Gasche R , Rubio A , Rennenberg H , Butterbach‐Bahl K , Kiese R . 2016 Nitrous oxide emissions from stems of ash (*Fraxinus angustifolia* Vahl) and European beech (*Fagus sylvatica* L.). Plant and Soil 398: 35–45.

[nph17002-bib-0012] Espenberg M , Truu M , Mander Ü , Kasak K , Nõlvak H , Ligi T , Oopkaup K , Maddison M , Truu J . 2018 Differences in microbial community structure and nitrogen cycling in natural and drained tropical peatland soils. Scientific Reports 8: 4742. 29549345 10.1038/s41598-018-23032-y PMC5856767

[nph17002-bib-0013] Gomez‐Alvarez V , King GM , Nüsslein K . 2007 Comparative bacterial diversity in recent Hawaiian volcanic deposits of different ages. FEMS Microbiology Ecology 60: 60–73. 17381525 10.1111/j.1574-6941.2006.00253.x

[nph17002-bib-0014] Hölttä T , Kolari P . 2009 Interpretation of stem CO_2_ efflux measurements. Tree Physiology 29: 1447–1456. 19773338 10.1093/treephys/tpp073

[nph17002-bib-0015] Jeffrey LC , Reithmaier G , Sippo JZ , Johnston SG , Tait DR , Harada Y , Maher DT . 2019 Are methane emissions from mangrove stems a cryptic carbon loss pathway? Insights from a catastrophic forest mortality. New Phytologist 224: 146–154. 10.1111/nph.15995 31211874

[nph17002-bib-0016] Keppler F , Hamilton JTG , Brass M , Röckmann T . 2006 Methane emissions from terrestrial plants under aerobic conditions. Nature 439: 187–191. 16407949 10.1038/nature04420

[nph17002-bib-0017] King GM . 2003 Contributions of atmospheric CO and hydrogen uptake to microbial dynamics on recent Hawaiian volcanic deposits. Applied and Environmental Microbiology 69: 4067–4075. 12839783 10.1128/AEM.69.7.4067-4075.2003 PMC165208

[nph17002-bib-0018] King GM , Nanba K . 2008 Distribution of atmospheric methane oxidation and methanotrophic communities on Hawaiian volcanic deposits and soils. Microbes and Environments 23: 326–330. 21558726 10.1264/jsme2.me08529

[nph17002-bib-0019] Kirman S . 2003 Cycles biogéochimiques et biodiversité en forêt tropicale humide: étude ďune succession primaire sur coulées basaltiques (La Réunion, Ocean Indien). PhD thesis, Aix‐Marseille University, Marseille, France.

[nph17002-bib-0020] Kirman S , Strasberg D , Grondin V , Colin F , Gilles J , Meunier JD . 2007 Biomass and litterfall in a native lowland rainforest: Mare Longue Reserve, La Réunion Island, Indian Ocean. Forest Ecology and Management 252: 257–266.

[nph17002-bib-0021] Kreft H , Jetz W , Mutke J , Kier G , Barthlott W . 2008 Global diversity of island floras from a macroecological perspective. Ecology Letters 11: 116–127. 18036182 10.1111/j.1461-0248.2007.01129.x

[nph17002-bib-0022] Lenhart K , Behrendt T , Greiner S , Steinkamp J , Well R , Giesemann A , Keppler F . 2019 Nitrous oxide effluxes from plants as a potentially important source to the atmosphere. New Phytologist 221: 1398–1408. 10.1111/nph.15455 30303249

[nph17002-bib-0023] Lenhart K , Weber B , Elbert W , Steinkamp J , Clough T , Crutzen P , Pöschl U , Keppler F . 2015 Nitrous oxide and methane emissions from cryptogamic covers. Global Change Biology 21: 3889–3900. 26152454 10.1111/gcb.12995

[nph17002-bib-0024] Machacova K , Bäck J , Vanhatalo A , Halmeenmäki E , Kolari P , Mammarella I , Pumpanen J , Acosta M , Urban O , Pihlatie M . 2016a *Pinus sylvestris* as a missing source of nitrous oxide and methane in boreal forest. Scientific Reports 6: 23410. 26997421 10.1038/srep23410 PMC4800674

[nph17002-bib-0025] Machacova K , Halmeenmäki E , Pihlatie M , Urban O . 2016b Seasonal courses of methane fluxes in boreal trees. Report Series in Aerosol Science 189: 308–311.

[nph17002-bib-0026] Machacova K , Maier M , Svobodova K , Lang F , Urban O . 2017 Cryptogamic stem covers may contribute to nitrous oxide consumption by mature beech trees. Scientific Reports 7: 13243. 29038453 10.1038/s41598-017-13781-7 PMC5643534

[nph17002-bib-0027] Machacova K , Papen H , Kreuzwieser J , Rennenberg H . 2013 Inundation strongly stimulates nitrous oxide emissions from stems of the upland tree *Fagus sylvatica* and the riparian tree *Alnus glutinosa* . Plant and Soil 364: 287–301.

[nph17002-bib-0028] Machacova K , Pihlatie M , Halmeenmäki E , Pavelka M , Dušek J , Bäck J , Urban O . 2015 Summer fluxes of nitrous oxide from boreal forest In: Urban O , Šprtová M , Klem K , eds. Global change: a complex challenge, conference proceedings. Brno, Czech Republic: Global Change Research Center, 78–81.

[nph17002-bib-0029] Machacova K , Vainio E , Urban O , Pihlatie M . 2019 Seasonal dynamics of stem N_2_O exchange follow the physiological activity of boreal trees. Nature Communications 10: 4989. 10.1038/s41467-019-12976-y PMC6825224 31676776

[nph17002-bib-0030] Maier M , Machacova K , Lang F , Svobodova K , Urban O . 2018 Combining soil and tree‐stem flux measurements and soil gas profiles to understand CH_4_ pathways in *Fagus sylvatica* forests. Journal of Plant Nutrition and Soil Science 181: 31–35.

[nph17002-bib-0031] Menyailo OV , Hungate BA . 2005 Tree species effects on potential production and consumption of carbon dioxide, methane, and nitrous oxide: the Siberian afforestation experiment In: Binkley D , Menyailo OV , eds. Tree species effects on soils: implications for global change. NATO Science Series. Dordrecht, the Netherlands: Kluwer Academic, 293–305.

[nph17002-bib-0032] Meunier JD , Kirman S , Strasberg D , Nicolini E , Delcher E , Keller C . 2010 The output and bio‐cycling of Si in a tropical rain forest developed on young basalt flows (La Reunion Island). Geoderma 159: 431–439.

[nph17002-bib-0033] Myhre G , Shindell D , Bréon F‐M , Collins W , Fuglestvedt J , Huang J , Koch D , Lamarque J‐F , Lee D , Mendoza B *et al* 2013 Anthropogenic and natural radiative forcing In: Stocker TF , Qin D , Plattner G‐K , Tignor M , Allen SK , Boschung J , Nauels A , Xia Y , Bex V , Midgley PM , eds. Climate Change 2013: The Physical Science Basis. *Contribution of Working Group I to the Fifth Assessment Report of the Intergovernmental Panel on Climate Change* Cambridge, UK & New York, NY, USA: Cambridge University Press, 659–740.

[nph17002-bib-0034] Pangala SR , Enrich‐Prast A , Basso LS , Peixoto RB , Bastviken D , Hornibrook ERC , Gatti LV , Ribeiro H , Calazans LSB , Sakuragui CM *et al* 2017 Large emissions from floodplain trees close the Amazon methane budget. Nature 552: 230–234. 29211724 10.1038/nature24639

[nph17002-bib-0035] Pangala SR , Moore S , Hornibrook ERC , Gauci V . 2013 Trees are major conduits for methane egress from tropical forested wetlands. New Phytologist 197: 524–531. 10.1111/nph.12031 23253335

[nph17002-bib-0036] Potgieter LJ , Wilson JRU , Strasberg D , Richardson DM . 2014 *Casuarina* invasion alters primary succession on lava flows on La Réunion Island. Biotropica 46: 268–275.

[nph17002-bib-0037] Richards PW . 1996 The tropical rain forest. Cambridge, UK: Cambridge University Press.

[nph17002-bib-0038] Rusch H , Rennenberg H . 1998 Black alder (*Alnus glutinosa* (L.) Gaertn.) trees mediate methane and nitrous oxide emission from the soil to the atmosphere. Plant and Soil 201: 1–7.

[nph17002-bib-0039] Saunois M , Bousquet P , Poulter B , Peregon A , Ciais P , Canadell JG , Dlugokencky EJ , Etiope G , Bastviken D , Houweling S *et al* 2016 The global methane budget 2000–2012. Earth System Science Data 8: 697–751.

[nph17002-bib-0040] Schindler T , Mander Ü , Machacova K , Espenberg M , Krasnov D , Escuer‐Gatius J , Veber G , Pärn J , Soosaar K . 2020 Short‐term flooding increases CH_4_ and N_2_O emissions from trees in a riparian forest soil–stem continuum. Scientific Reports 10: 3204. 32081925 10.1038/s41598-020-60058-7 PMC7035275

[nph17002-bib-0041] Shvidenko A , Barber CV , Persson R , Gonzalez P , Hassan R , Lakyda P , McCallum I , Nilsson S , Pulhin J , van Rosenburg B *et al* 2005 Forest and woodland systems In: Hassan R , Scholes R , Ash N , eds. Ecosystems and human well‐being: current state and trends. Washington, DC, USA: Island Press, 585–621.

[nph17002-bib-0042] Sjögersten S , Siegenthaler A , Lopez OR , Aplin P , Turner B , Gauci V . 2020 Methane emissions from tree stems in neotropical peatlands. New Phytologist 225: 769–781. 10.1111/nph.16178 PMC6973267 31495939

[nph17002-bib-0043] Smart DR , Bloom AJ . 2001 Wheat leaves emit nitrous oxide during nitrate assimilation. Proceedings of the National Academy of Sciences, USA 98: 7875–7878. 10.1073/pnas.131572798 PMC35435 11427711

[nph17002-bib-0044] Smith KA , Ball T , Conen F , Dobbie KE , Massheder J , Rey A . 2003 Exchange of greenhouse gases between soil and atmosphere: interactions of soil physical factors and biological processes. European Journal of Soil Science 54: 779–791.

[nph17002-bib-0045] Sundqvist E , Crill P , Mölder M , Vestin P , Lindroth A . 2012 Atmospheric methane removal by boreal plants. Geophysical Research Letters 39: L21806.

[nph17002-bib-0046] Turner IM , Corlett RT . 1996 The conservation value of small, isolated fragments of lowland tropical rain forest. Tree 11: 330–333. 21237864 10.1016/0169-5347(96)10046-x

[nph17002-bib-0047] Warlo H , Machacova K , Nordstrom N , Maier M , Laemmel T , Roos A , Schack‐Kirchner H . 2018 Comparison of portable devices for sub‐ambient concentration measurements of methane (CH_4_) and nitrous oxide (N_2_O) in soil research. International Journal of Environmental Analytical Chemistry 98: 1030–1037.

[nph17002-bib-0048] Welch B , Gauci V , Sayer EJ . 2018 Tree stem bases are sources of CH_4_ and N_2_O in a tropical forest on upland soil during the dry to wet season transition. Global Change Biology 25: 361–372. 30367532 10.1111/gcb.14498

[nph17002-bib-0049] Wen Y , Corre MD , Rachow C , Chen L , Veldkamp E . 2017 Nitrous oxide emissions from stems of alder, beech and spruce in a temperate forest. Plant and Soil 420: 423–434.

[nph17002-bib-0050] Yu K , Chen G . 2009 Nitrous oxide emissions from terrestrial plants: observations, mechanisms and implications In: Sheldon AI , Barnhart EP , eds. Nitrous oxide emissions research progress. Hauppauge, NY, USA: Nova Science Publishers, 85–104.

