# Supplementary material for: Trees as net sinks for methane (CH4) and nitrous oxide (N2O) in the lowland tropical rain forest on volcanic Réunion Island
Source: New Phytol. 2020 Nov 18;229(4):1983–94. doi: 10.1111/nph.17002 (PMC7894294; doi:10.1111/nph.17002)
Supplement: Supplementary file 1 — Fig. S1 Examples of CH4, N2O and CO2 concentration changes over time in the headspaces of soil chambers, volcanic rock chambers, tree stem chambers and incubation chambers containing cryptogams. Please note: Wiley Blackwell are not responsible for the content or functionality of any Supporting Information supplied by the authors. Any queries (other than missing material) should be directed to the New Phytologist Central Office. [file NPH-229-1983-s001.pdf]

New Phytologist

***New Phytologist* Supporting Information**

Article title: **Trees as net sinks for methane (CH_4) and nitrous oxide (N_2O) in the lowland tropical rain forest on volcanic Réunion Island**

Authors: Katerina Machacova[1,*], Libor Borak[1], Thomas Agyei[1], Thomas Schindler[1,2], Kaido Soosaar[1,2], Ülo Mander[1,2], Claudine Ah-Peng[3]

Article acceptance date: 01 October 2020

The following Supporting Information is available for this article:

Fig. S1 Examples of CH_4 (a, d, g, j), N_2O (b, e, h, k) and CO_2 (c, f, i, l) concentration changes over time in headspace of soil chambers (a, b, c; soil position close to *Homalium paniculatum*), volcanic rock chambers (d, e, f), tree stem chambers (g, h, i; *Homalium paniculatum*) and incubation chambers containing cryptogams (j, k, l; *Pyrrhobryum spiniforme*). The gas concentrations were assessed by portable greenhouse gas analyzer. Decrease of gas concentration indicates gas uptake, increase of gas concentration indicates gas emission.

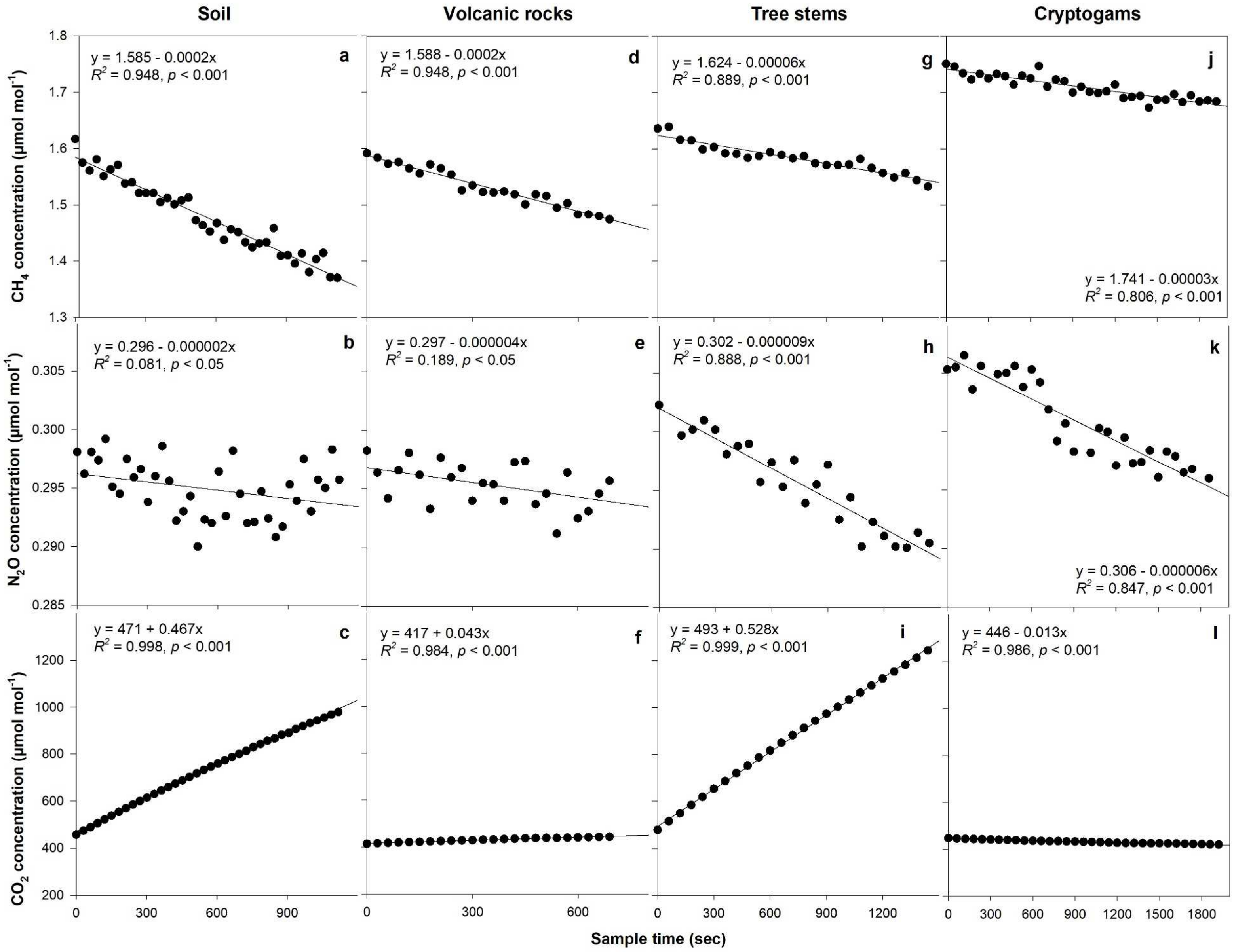

Soil
Volcanic rocks
Tree stems
Cryptogams
y = 1.585 - 0.0002x
R2 = 0.948, p < 0.001
a
y = 1.588 - 0.0002x
R2 = 0.948, p < 0.001
d
y = 1.624 - 0.00006x
R2 = 0.889, p < 0.001
g
j
y = 1.741 - 0.00003x
R2 = 0.806, p < 0.001
y = 0.296 - 0.000002x
R2 = 0.081, p < 0.05
b
y = 0.297 - 0.000004x
R2 = 0.189, p < 0.05
e
y = 0.302 - 0.000009x
R2 = 0.888, p < 0.001
h
k
y = 0.306 - 0.000006x
R2 = 0.847, p < 0.001
y = 471 + 0.467x
R2 = 0.998, p < 0.001
c
y = 417 + 0.043x
R2 = 0.984, p < 0.001
f
y = 493 + 0.528x
R2 = 0.999, p < 0.001
i
y = 446 - 0.013x
R2 = 0.986, p < 0.001
l
CH4 concentration (µmol mol-1)
N2O concentration (µmol mol-1)
CO2 concentration (µmol mol-1)
Sample time (sec)
